# Perception of work in the IT sector among men and women—A comparison between IT students and IT professionals

**DOI:** 10.3389/fpsyg.2022.944377

**Published:** 2022-09-09

**Authors:** Joanna Pyrkosz-Pacyna, Karolina Dukala, Natasza Kosakowska-Berezecka

**Affiliations:** ^1^Faculty of Humanities, AGH University of Science and Technology, Kraków, Poland; ^2^Faculty of Psychology in Katowice, SWPS University of Social Sciences and Humanities, Katowice, Poland; ^3^Faculty of Social Sciences, University of Gdansk, Gdańsk, Poland

**Keywords:** STEM, IT, gender, work interests, work perception

## Abstract

Lack of gender balance within STEM fields is caused by many complex factors, some of which are related to the fact that women do not perceive certain occupations as congruent with their career and personal goals. Although there is a large body of research regarding women in STEM, there is a gap concerning perception of occupations within different STEM industries. IT is a domain where skilled employees are constantly in demand. Even though the overall female representation in STEM fields is rising and that the IT industry is undertaking numerous interventions to attract women to careers in IT, the representation of women in this domain is still disappointingly low. Therefore, the goal of our study was to examine the possible differences among male and female IT and non-IT students and employees in terms of their perception of IT and other key factors influencing the feeling of aptness of IT as a potential sector one's career: goal congruence, sense of belonging and self-efficacy. In this paper we present the results of a study conducted in Poland among working IT professionals (*N* = 205) and IT students (*N* = 127) that we compare with individuals from non-IT sectors (*N* = 222 employees, 107 students). Our results showed significant gender differences between IT students and IT professionals. We found that communal goals are more important for IT employees than for IT students (both male and female) and that a sense of social belonging is stronger among female IT employees than among male IT employees and IT students. Women employed in IT also had the same level of sense of social belonging as women in non-IT group. These findings suggest that after entering IT positions, women's perception of the domain might become potentially more favorable and attuned with their needs. We also found that female IT students value agentic goals more than communal goals which was not the case for female IT employees. The results highlight the importance of investigating women's perception of the IT sector at different levels of career in terms of their goals and other work-related variables. Such lines of research will help develop more effective interventions in attracting women to enter the IT field.

## Introduction

Despite numerous interventions aimed at reaching gender equality in STEM (Science, Technology, Engineering, Mathematics) domains women are still vastly underrepresented in many STEM-related professions. STEM represents multiple and very distinct technological disciplines among which some are more, and some are less represented by women. Among the industries most highly dominated by men is IT (Information Technology). The proportion of women in IT in USA is reported to be ~25% (Ashcraft et al., 2016; Fry et al., 2021), with more detailed data from 2020 showing that women make up 28–42% of the GAFAM (Google, Apple, Facebook, Amazon, and Microsoft; Statista, 2021). Nevertheless these women who enter IT jobs, are less likely than men to work as programmers and tend to occupy positions of a tester or project manager. According to the Women in IT (2022) 70% of IT female specialists reported difficulties in entering this line of work. Similarly to the labor market, within academia unequal representation of women and men is visible. Among all STEM majors, IT manifests one of the largest gender gaps. In domains such as mathematics, physics, or life-sciences the female to male students' ratio is less unbalanced (Fry et al., 2021). Yet in IT the change in female representation over the years is barely visible (Jasko et al., 2020; European Commission Directorate-General for Research and Innovation, 2021; Fry et al., 2021). For example, the Women in Polytechnics Report (Knapińska, 2022) shows that the largest ever increase in the number of female IT students in Poland recorded the historical spike from 12% in 2014 to 14% in year 2018. Authors of this report argue that this spike in admissions might stem from multiple initiatives and interventions aimed at women in IT such as “IT for SHE,” “Geek Girls Carrots,” “Girls in Tech” or “Women in Technology”. This is however not an informed conclusion as to our best knowledge, no data is available proving effectiveness of programs in enhancing women's intention to enter and remain in IT sector.

There are however some results available showing the effectiveness of interventions aimed at more equal gender representation in STEM. For example, Dennehy and Dasgupta (2017) showed in the longitudinal experiment, that having a female peer mentor early in college increases women's positive academic experiences and retention in engineering. In the study by Ramsey et al. (2013) interventions focused on creating a welcoming academic environment resulted in decreased stereotyping concerns and increased implicit STEM identification among STEM female students. Interestingly, there is also evidence for unsuccessful interventions. Cowgill et al. (2021) for example demonstrated how programs overtly emphasizing women's minority status in STEM might lead to opposite effects such as decrease in women's interest in STEM. It is important to mention at this point, that all the above results pertain to interventions conducted with the US samples. The data from other regions is scarce. For example in a study by Peña et al. (2021) focusing on diagnosing the extent to which gender issues were even mentioned within STEM teaching programs, 84% of the teaching staff stated that their proposed activities did not include any gender aspects. It is also worth noting that these scarce interventions that are rarely evaluated usually pertain to STEM as a discipline in general—to our knowledge there are no domain specific analyses that would focus on IT only.

Previous literature provided explanations for possible reasons for low female representation in STEM among university majors and among employees (for example: Dweck, 2007; Cheryan et al., 2009; Diekman et al., 2010; Singh et al., 2013). Less is known about women particularly in the IT field. It seems to be a unique domain where on the one hand women are constantly on the margins and on the other hand where the demand for highly qualified staff is continuously rising (United Nations Technology and Innovation Labs Report, 2019; Fry et al., 2021). What is more, most of the research undertaking the topic of women's lower representation in STEM has been focused on western samples, with fewer studies conducted within the context of Eastern and Central Europe. Our study thus contributes to current research lines focused on lower female representation in the IT sector by adding another under-researched context that of Poland.

Drawing on the well-established theoretical approaches (i.e., Goal Congruence Theory, Self-Efficacy and Sense of Social Belonging) we carried out an empirical research exploring the factors most relevant to exclusion of girls and women from STEM, specifically the field of IT. Our research contributes to finding solutions for low representation of women in the highly male-dominated sector of IT by focusing on perception of this domain among men and women. We have also looked at different stages of the career path within IT by including both IT students and IT employees in our sample. Specifically we examine the possible differences among male and female IT and non-IT students and employees in terms of their perception of IT and other key factors potentially influencing the feeling of aptness of IT: goal congruence, sense of belonging and self-efficacy. We examine women's perceptions of IT while they are IT students and when they are IT professionals. Comparing women at two different career stages allows us to verify how the potential personal misfit to IT may be detrimental for women who comply with commonly accepted social norms. Such lines of research help define areas in which women are mostly vulnerable to gender stereotypes and thus decide to quit this career.

One of the factors contributing largely to low female representation in STEM domains is the lack of perceived goal congruence. Women tend to prioritize communal goals and therefore might discard IT as a domain that does not afford those goals – as a result they don't feel they belong to the IT sector. Additionally, women in STEM experience self-efficacy decline along their career progress (Brainard and Carlin, 1998; Sterling et al., 2020; Stewart et al., 2020) which, when combined with the conviction that IT requires high intellectual capabilities, discourages them from entering this career field. This may suggest why women, even when skilled in other scientific matters, opt out from choosing a career in IT as early as when choosing their study majors.

### Women in IT sector: Goal congruence

In recent years researchers provided valuable insights into possible mechanisms contributing to low female representation in STEM, which can be crucial when analyzing the situation of women in the IT sector. The obtained results referred to in the literature highlight the importance of analyzing how women perceive a given sector as allowing them to realize their career goals. The Goal Congruence Theory (Diekman et al., 2010; Brown et al., 2015; Steinberg and Diekman, 2017) focuses on two categories of people's goals: communal (i.e. working with others, helping others or cooperation) and agentic (i.e. power, status, career development or expertise growth). It tackles two aspects of these goals: goal endorsement (the type of goals that are personally important and are targeted for pursuit) and goal affordance (perception of possibility to achieve certain goals) both of which may influence the decision to engage in certain domains. In a series of studies, Diekman et al. (2010) showed that STEM occupations are perceived as affording communal goals to a lesser extent than other jobs and that this perception among women in turn influences the decisions regarding future career orientation. Since women tend to lean toward occupations that afford communal goals congruence, the perception of STEM might be one of the key reasons for low female representation in those domains. These results are especially valid for the IT industry as it might be perceived as a technical and solitary type of occupation. Numerous studies point out the existence of specific IT specialist stereotypes that entail isolation, social inadequacy, over-focus on technology, unpopularity, and most importantly, masculinity (Herz, 1997; Sanger et al., 1997; Clayton et al., 2009; Santos et al., 2022). This might deter women choosing IT as their career path.

On the other hand, analyses of possible indicators of high female drop-out ratio show the importance of not only communal goals endorsement but also the need to meet their agentic goals. An increasing number of studies show that agentic goals are becoming equally important for men as well as for women (Moore et al., 2008; Pyrkosz-Pacyna et al., 2019). Yet satisfying agentic goals (in women's case) in IT is not as evident as one may expect. Fouad et al. (2012) found that reduced access to key creative roles, and a sense of feeling stalled in one's career are among others the most significant factors contributing to female attrition from the tech field. Additionally, women who left IT positions were less likely to report opportunities for training and development and support from a manager within their former workplace. It would therefore seem that even though IT is perceived as an agentic sector, women in IT are uncertain if they can attain their agentic goals. In turn, lack of goal congruence might impact another important variable relating to women's intention to stay within the IT sector both when they are students and when they are professionals—that variable is the sense of social belonging.

### Sense of social belonging and women's representation in the IT sector

The sense of social belonging is the conviction that one is accurately fitting into a given environment. It also entails perception of social connectedness in groups and the sense of fitting in socially with others (Baumeister and Leary, 1995; Walton and Cohen, 2007). Numerous studies have explored the importance of social belonging in reference to women's representation in STEM. These studies (Cheryan and Plaut, 2010; Good et al., 2012; Tellhed et al., 2017; Aelenei et al., 2020) showed that indeed women tend to feel that they do not naturally belong in STEM and conversely, by experimentally increasing their sense of fit, women declared more interest in career in STEM. Very subtle cues can make the shift away or toward STEM. For example, in a study by Cheryan et al. (2009) altering the surroundings of college career advisory meetings (reflecting stereotypical tech-geek space: sci-fi posters, video games stations or empty energy drink cans vs. neutral one) influenced the women's willingness to consider IT as a college major. Even women already working in IT notice the specific image of this industry, admitting that the “geekiness” is a part of the IT image (Moore et al., 2008).

At the same time, there is evidence that despite the possible lowered sense of belonging women feel the connection with the IT field. The study conducted at Technological University (Pyrkosz-Pacyna et al., 2019) revealed that women's experience of a sense of belonging to the University was the same as that of men. The existing evidence is scarce and mixed. It requires further analyses that would allow us to explore the level of women's sense of belonging to the IT sector at different career levels.

### Women's self-efficacy in the IT sector

According to the value - expectancy theory (Eccles, 1987) people tend to engage in endeavors that are viewed as both valuable and reachable. The classic study by Bandura (1997) showed that self-efficacy, that can be defined as person's belief in his or her capability to successfully perform a particular task, is related to the quantity of required effort and the willingness to persist at tasks. If the goal is perceived as outside one's competences, the action toward it might be weak or forsaken altogether. The perception of one's own capabilities plays an important role in considering various career choices. Numerous studies showed that women in general tend to have lower self-efficacy especially in STEM fields, even when controlling for the actual educational outcomes (Correll, 2001; Singh et al., 2007; Good et al., 2012; Sterling et al., 2020). Additionally, science is perceived as a domain where those with exceptional innate talents are more frequently represented (Dweck, 2007). Women also tend to hold themselves to much higher standards when it comes to what they perceive as high skills (Correll, 2004), and they report lower self-efficacy than men regardless of their actual performance (Kost-Smith et al., 2009; Stout et al., 2011). As a result, their self-efficacy convictions are strongly related to engagement in STEM. Studies showed that low math self-efficacy predicts low intention to pursue a STEM career (Correll, 2001) and similarly the computer self-efficacy (Miura, 1987, 2020). It is important to highlight that self-efficacy is not necessarily representative of one's actual skills. There is robust evidence showing that there are no observable gender differences in STEM competences (U.S. Department of Education, 2007) yet the self-perception of those skills is continuously biased, with men assessing their science skills more positively than women (Correll, 2001; Stewart et al., 2020). The bias against women in the IT sector also plays a role here. The stereotypes that women are less skilled in programming tasks can cause great damage. Studies by Terrell et al. (2016) showed that codes prepared by men and by women were assessed equally, but only if the coder gender was not revealed. Yet, as in the case of previous factors, further studies are needed to explore the perception of women's skills in the IT sector, which in turn has a significant impact on their engagement in this domain.

In our research we focus on two groups of women: (1) women who have chosen IT as their major and (2) those who are employed in IT companies. We compare those results to the results of men and to comparable samples of women studying or working in non-IT sectors. This allows us to gather data about women at different stages of their IT career trajectory. The transition from university to employment is a unique time period. Data shows that even women who graduated from IT majors are likely to forgo their career in this field (Gu, 2018) which is a huge waste of their previous efforts and their unique vocational potential. Our sample consisting of middle and eastern European participants is also uniquely valuable as Poland is a country with considerable IT outsourcing facilities. The market for IT employees in Poland is significant (Kossowska et al., 2012). Results of our study contribute to the knowledge about factors significant in designing and implementing various interventions for women in STEM with specific focus on the IT field.

### The present research

To explore possible differences between perception of future work conditions and the actual experience of employees in the IT sector and inform future interventions, our sample consists of (male and female) IT students and IT employees. To make sure that these findings are specific to the IT field, we have included a comparison group composed of non-IT women, including management students and banking employees (male and female) that do not belong to STEM fields. The obtained results can contribute to designing effective interventions both specifically in central European setting and in general by showing how work in IT is perceived in multiple perspectives: that of men and women, on different levels of their career trajectory and in comparison to other domains. The knowledge of various factors impacting attitudes toward work in IT can be applied in designing interventions based on familiarity with the targeted group of interest. For example, perhaps different interventions might be suitable for women in IT jobs than for IT students when the intervention is tackling sense of social belonging to the domain. Detailed hypotheses are described below.

#### Goal endorsement and goal affordance hypotheses

We predicted that women in IT will value communal goals more than men in IT (H 1a) but less than women working in non-IT fields (H 1b). Since IT is perceived as mostly affording agentic goals, we suspected those women who decided to go into IT will value these goals more than women in non-IT sample (H 1c). We also overall predicted that women in IT will value communal goals more than agentic goals (H 1d). In our study we also wanted to test whether there are any differences between female IT employees and IT students. These analyses were exploratory, along with between-field, IT vs non-IT comparisons.

When it comes to perception of IT in terms of goal affordance, we predicted that women in IT will perceive lower goal affordance, both communal (H 2a) and agentic (H 2b) than men, and then women in non-IT working environments (H 2c and H 2d respectively). As before we also explored differences between female IT employees and students and across fields (IT vs. non-IT).

#### Social belonging hypotheses

Since IT is dominated by men and is stereotypically considered as a manly profession, we predicted that women in IT will declare a lower sense of social belonging than men in IT (H 3a) and non-IT women (H 3b). Again, we explored the differences between female IT employees and students and across IT and non-IT fields.

#### Self-efficacy hypotheses

We predicted, according to existing literature, that women in IT will have lower self-efficacy than men in IT (H 4a) and then non-IT professional women due to perceiving IT as a domain where special skills usually connected to masculinity are needed (H 4b). We also explored the differences among female IT employees and students regarding their self-efficacy. Again, we explored the differences across fields.

## Method

### Procedure

The study was conducted in early 2019 (before COVID-19 pandemic), among IT (informatics) majors and management students and among IT and management professionals. IT and management students were contacted via email with a link to the survey attached. For taking part in the study, participants were offered the possibility to participate in a draw to receive a gratuity in the form of a university gadgets gift box (containing fountain pen or USB drive). For the sake of anonymity participants were informed that to take part in the draw they should provide their contact information in a separate link available at the end of the survey, so it will be impossible to link their results with personal information. Participants could fill in the questionnaires only in an online form, in their free time.

Professionals were recruited from two companies at their Polish divisions: an international banking corporation (with an expanded IT department) and an IT company. We wanted to include a sample from non-STEM and at the same time not stereotypically female industry and banking/management is a good example of such industry. The invitation to take part in the study was sent out by the internal communication system to both IT and non-IT departments of the companies. Participants were not offered any remuneration for taking part in the study. Since one of the companies where we conducted the study is international and hires international staff, upon the request of the company representatives, the survey was also available in English for non-Polish participants. However, due to the very small sample size, we have excluded answers of English speaking participants from further analysis. Participants filled in online questionnaires while at work.

### Participants

A total sample of 724 participants took part in the study. 63 surveys were dropped because participants did not indicate their gender or were outside the IT/banking sector, so 661 surveys were used for further analysis. Final sample consisted of 127 IT students (73 female), 107 control group students (57 female), 205 IT employees (82 female) and 222 control group employees (137 female). Mean age of employees was *M* = 31.04 (*SD* = 6.08) and *M* = 22.64 (*SD* = 3.59) of students. 180 students were on the bachelor's degree level and 54 were master's degree students.

In the sample of employees mean seniority in the company was M = 3.27, SD = 3.13, minimum 0 years and maximum 26 years.

### Measures

All scales were back translated into Polish by a native speaker. All items were adjusted for employees/students participants (e.g., “Do you feel confident about your line of work?” for employees and “Do you feel confident about your line of studies?” for students).

#### Goal congruence

Scale developed by Diekman et al. (2010). The scale consists of 23 goals in two dimensions: 14 on agency (e.g., achievement, power) and 9 on communality (e.g., helping others, serving humanity). Using a scale from 1 to 7, participants were asked to determine how much each goal is personally important for them (goal endorsement) and how much their work domain (or major in the case of students) allows them to achieve those goals (goal affordance), where 1 = unimportant/ this domain does not enable achieving this goal, and 7 = very important/ this domain enables achieving this goal. Reliability of the scale was Cronbach's α = 0.840 for agency endorsement, Cronbach's α = 859, communal endorsement, Cronbach's α = 0.916 for agency affordance and Cronbach's α = 0.836 for communal affordance (all coefficients were computed for the whole sample, both employees and students).

#### Self-efficacy

Scale developed by Dennehy and Dasgupta (2017), consist of 6 items (e.g., Do you think you have a talent for your line of work? Do you feel confident about your line of work?) on 1 (I disagree) to 7 (I agree) Likert scale (Cronbach's α = 0.703).

#### Sense of social belonging

Scale developed by Dennehy and Dasgupta (2017) consist of four statements, e.g., “I feel connected to my colleagues in my field” and measures an individual's sense of belonging to the given field, with 1 to 7 scale (1 = I disagree, 7 = I agree) (Cronbach's α = 0.826).

### Analysis strategy

The goal of our analysis was to show the specificity of the situation of women in the IT field, therefore we compared women's results to (1) men working in the IT sector and to (2) women working outside of the IT sector. We later compared IT female employees with IT students' results. Hence, analyses of all dependent variables are mostly conducted in three steps: in step 1 we compare male and female employees in the IT field (MANOVA with gender as an IV, we present those analyses in Table 1). In step 2 we compare female employees and students in the IT vs. non-IT field (MANOVAs with area of work as an IV, these analyses are presented in Table 2). In the last step we compare both IT female employees and IT female students and IT male employees to IT male students (MANOVAs with work status as IV, all those analyses are presented in Table 3). Other analyses are mentioned in the text. Other information as well as the syntax and database can be found in the supplementary materials. All correlations between variables are in Table 4. Means, confidence intervals and the differences between means in all groups are in Table 5.

**Table 1:** Results of a series of MANOVAs showing differences between male vs. female employees and male vs. female students in the IT field.

	**IT employees**							**IT students**						
	**Female**		**Male**					**Female**		**Male**				
	***M***	***SD***	***M***	***SD***	***F*_(1,203)_**	***p***	***η^2^***	***M***	***SD***	***M***	***SD***	***F*_(1,127)_**	***p***	***η^2^***
1. Communal endorsement	5.04	1.04	4.64	1.15	6.48*	0.012	0.03	4.36	1.07	3.99	1.09	3.66	0.058	0.03
2. Agentic endorsement	5.14	0.71	5.13	0.75	0.01	0.905	0.01	5.12	0.69	5.09	0.79	0.06	0.799	0.01
3. Communal affordance	4.61	1.07	4.34	1.18	2.92	0.089	0.01	4.10	0.92	4.03	1.03	0.22	0.693	0.01
4. Agentic affordance	4.84	0.97	4.67	1.03	1.48	0.225	0.01	5.78	0.65	5.35	0.93	9.15*	0.003	0.07
5. Social belonging	5.87	0.96	5.44	1.22	7.16*	0.008	0.03	5.08	1.45	5.31	1.28	0.87	0.352	0.01
6. Self-efficacy	5.30	0.84	5.22	0.91	0.33	0.566	0.01	4.26	0.87	4.61	0.95	4.77*	0.031	0.04

* p <0.05. This table corresponds with hypotheses H 1a, H 1c, H 2a, H 2b, H 3a, H 4a.

**Table 2:** Results of a series of MANOVAs showing differences between female employees and students in IT vs. non-IT fields.

**Dependent variable**	**Female employees**							**Female students**						
	**IT field**		**Non-IT field**					**IT field**		**Non-IT field**				
	***M***	***SD***	***M***	***SD***	***F*_(1,217)_**	***p***	***η^2^***	***M***	***SD***	***M***	***SD***	***F*_(1,128)_**	***p***	***η^2^***
1. Communal endorsement	5.04	1.04	5.06	0.99	0.02	0.880	0.01	4.36	1.07	4.97	1.21	9.14*	0.003	0.07
2. Agentic endorsement	5.14	0.70	5.27	0.78	1.52	0.219	0.01	5.12	0.69	5.38	0.92	3.46	0.065	0.03
3. Communal affordance	4.61	1.07	4.34	1.22	2.77	0.097	0.01	4.10	0.92	4.51	1.16	5.24*	0.024	0.04
4. Agentic affordance	4.84	0.97	4.52	1.02	5.46*	0.020	0.03	5.78	0.65	5.33	1.11	8.26*	0.005	0.06
5. Social belonging	5.87	0.96	5.59	1.23	2.95	0.087	0.01	5.08	1.45	5.44	1.46	1.96	0.164	0.01
6. Self-efficacy	5.30	0.84	5.29	0.92	0.01	0.993	0.01	4.26	0.87	4.50	1.16	1.80	0.182	0.01

* p <0.05. This table corresponds with hypotheses H 1b, H 2c, H 2d. H 3b, H 4b.

**Table 3:** Results of a series of MANOVAs showing differences between employees and students in the IT field.

**Dependent variable**	**IT female**							**IT male**						
	**Employees**		**Students**					**Employees**		**Students**				
	***M***	***SD***	***M***	***SD***	***F*_(1,153)_**	***p***	***η^2^***	***M***	***SD***	***M***	***SD***	***F*_(1,175)_**	***p***	***η^2^***
1. Communal endorsement	5.04	1.04	4.36	1.07	16.13**	<0.001	0.10	4.64	1.15	3.99	1.09	12.59**	<0.001	0.07
2. Agentic endorsement	5.14	0.70	5.11	0.69	0.03	0.858	<0.01	5.13	0.75	5.09	0.79	0.11	0.741	0.01
3. Communal affordance	4.61	1.07	4.10	0.92	10.30*	0.002	0.06	4.34	1.18	4.03	1.03	2.79	0.097	0.02
4. Agentic affordance	4.84	0.97	5.78	0.65	48.32**	<0.001	0.24	4.67	1.03	5.35	0.93	17.49**	<0.001	0.09
5. Social belonging	5.87	0.96	5.08	1.45	16.16**	<0.001	0.10	5.44	1.22	5.31	1.28	0.39	0.531	0.01
6. Self-efficacy	5.30	0.84	4.26	0.87	56.22**	<0.001	0.27	5.22	0.91	4.61	0.94	16.41**	<0.001	0.09

* p < 0.05 and

** p < 0.001. This table corresponds with exploratory analysis.

**Table 4:** Means, standard deviations and correlations between main variables.

**Variable**	***M***	***SD***	**1**	**2**	**3**	**4**	**5**
1. Agentic endorsement	5.20	0.81	–				
2. Communality endorsement	4.72	1.11	0.124**	–			
3. Agentic affordance	4.93	1.08	0.233**	0.050	–		
4. Communality affordance	4.33	1.15	0.195**	0.372**	0.415**	–	
5. Self-efficacy	4.97	1.00	0.236**	0.207**	0.113**	0.265**	–
6. Belonging	5.49	1.28	0.080*	0.118**	0.209**	0.249**	0.489**

* p < 0.05,

** p < 0.001.

**Table 5:** Means and confidence intervals for all dependent variables for female and male employees and students in IT and business fields.

	**IT employees**		**Business employees**		**IT students**		**Business students**	
	***M _*female*_***	***M _*male*_***	***M _*female*_***	***M _*male*_***	***M _*female*_***	***M _*male*_***	***M _*female*_***	***M _*male*_***
	**[95% CI]**	**[95% CI]**	**[95% CI]**	**[95% CI]**	**[95% CI]**	**[95% CI]**	**[95% CI]**	**[95% CI]**
1. Communal endorsement	5.05ad	4.64bd	5.06a	4.71bd	4.36bc	3.99c	4.97d	4.52d
	[4.81, 5.27]	[4.46, 4.84]	[4.09, 5.24]	[4.48, 4.93]	[4.11, 4.61]	[3.70, 4.28]	[4.68, 5.25]	[4.22, 4.82]
2. Agentic endorsement	5.14ab	5.13ab	5.27ab	5.12a	5.11ab	5.09ab	5.38ab	5.48ab
	[4.98, 5.30]	[5.00, 5.26]	[5.12, 5.49]	[4.93, 5.13]	[4.93, 5.30]	[4.88, 5.30]	[5.17, 5.59]	[5.26, 5.69]
3. Communal affordance	4.61a	4.34ac	4.34abc	4.16bc	4.10c	4.03c	4.51d	4.62d
	[4.35, 4.88]	[4.12, 4.55]	[4.14, 4.55]	[3.90, 4.42]	[3.87, 4.33]	[3.76, 4.30]	[4.25, 4.78]	[4.34, 4.90]
4. Agentic affordance	4.84a	4.67a	4.52a	4.62a	5.78b	5.35c	5.33c	5.24c
	[4.62, 5.06]	[4.90, 4.85]	[4.33, 4.70]	[4.39, 4.86]	[5.56, 5.98]	[5.12, 5.59]	[5.10, 5.56]	[4.99, 5.48]
5. Social belonging	5.87a	5.44b	5.59ab	5.39b	5.08b	5.31b	5.44b	5.79a
	[5.61, 6.13]	[5.22, 5.64]	[5.39, 6.13]	[5.13, 5.64]	[4.76, 5.40]	[4.94, 5.40]	[5.08, 5.80]	[5.41,6.17]
6. Self-efficacy	5.30a	5.22ac	5.29a	5.13a	4.26b	4.61c	4.50bc	4.61bc
	[5.10, 5.49]	[5.07, 5.38]	[5.14, 5.45]	[4.93, 5.33]	[4.05, 4.47]	[4.37, 4.86]	[4.23, 4.77]	[4.32, 4.90]

Values in square brackets indicate the 95% confidence interval for each mean, lower-limit and upper-limit, respectively. The small letters indicate the differences between means.

## Results

### Goal endorsement

First, we tested if women in IT will value communal goals more than men in IT (Hypothesis 1a). We observed a significant difference [*F*_(1,203)_ = 6.48, *p* = 0.012, η^2^ = 0.03]: female employees in IT valued communal goals more than male. We observed a lack of effect for female students in IT compared to male students [*F*_(1,127)_ = 3.66, *p* = 0.058, η^2^ = 0.03].

Contrary to our hypothesis 1b, female employees in IT rated communal goal endorsement at the same level as women outside of IT [*F*_(1,217_) = 0.02, *p* = 0.880, η^2^ < 0.01]. A different pattern was observed for female students [*F*_(1,128)_ = 9.14, *p* = 0.003, η^2^ = 0.07]: they perceived communal goal endorsement at a lower level than women studying outside of IT.

Similarly, male employees in IT rated communal goals at the same level as men in the comparison group [*F*_(1,206)_ = 0.19, *p* = 0.664, η^2^ = 0.01], and male students in IT perceived communal goals at a lower level than students outside IT [*F*_(1,102)_ = 7.17, *p* = 0.009, η^2^ = 0.07].

In the next step we were interested in perception of agentic goals. We investigated the difference between male and female IT employees, and we found no difference in terms of agentic goals for female and male employees [*F*_(1,203)_ = 0.01, *p* = 0.905, η^2^ = 0.01] and students [*F*_(1,125)_ = 0.06, *p* = 0.799, η^2^ = 0.01].

Next, we checked the difference between women employees in IT vs. non-IT in terms of perception of agentic goals (Hypothesis 1c). We observed no significant difference [*F*_(1,217)_ = 1.52, *p* = 0.219, η^2^ = 0.01], the same lack of effect was for female students [*F*_(1,128)_ = 3.46, *p* = 0.065, η*2* = 0.03]. There were also no differences for male IT vs. non-IT employees in terms of importance of agentic goals [*F*_(1,206)_ = 0.01, *p* = 0.986, η^2^ = 0.01], but male IT students had lower agentic goal endorsement than male students outside IT [*F*_(1,102)_ = 6.80, *p* = 0.011, η^2^ = 0.06].

Finally, we tested if women in IT value communal goals more than agentic goals (Hypothesis 1d). We conducted repeated measures ANOVA only for women in IT, with the importance of communal and agentic goals as dependent variables. The difference between the importance of the two types of goals was not significant [*F*_(1,81)_ = 0.53, *p* = 0.469, η^2^ <0.01]. Female employees in IT value agentic and communal goals at the same level as female employees outside of IT. The pattern was different for IT female students. Female students in IT value agentic goals more than communal goals [*F*_(1,72)_ = 10.47, *p* = 0.002, η^2^ = 0.127]. On the other hand, men in IT rated agentic goals higher than communal goals, which was true for employees [*F*_(1,122)_ = 18.38, *p* <0.001, η^2^ = 0.13] and students [*F*_(1,53)_ = 26.17, *p* <0.001, η^2^ = 0.35].

**Figure 1:**
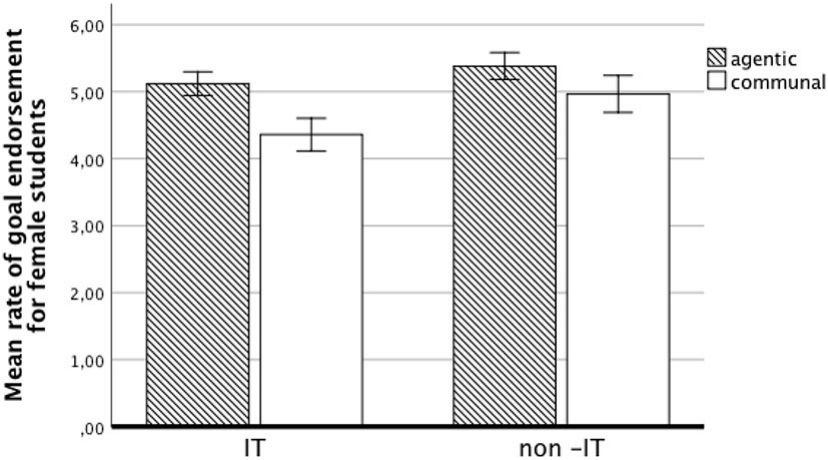
Mean rate of goal endorsement for female students.

**Figure 2:**
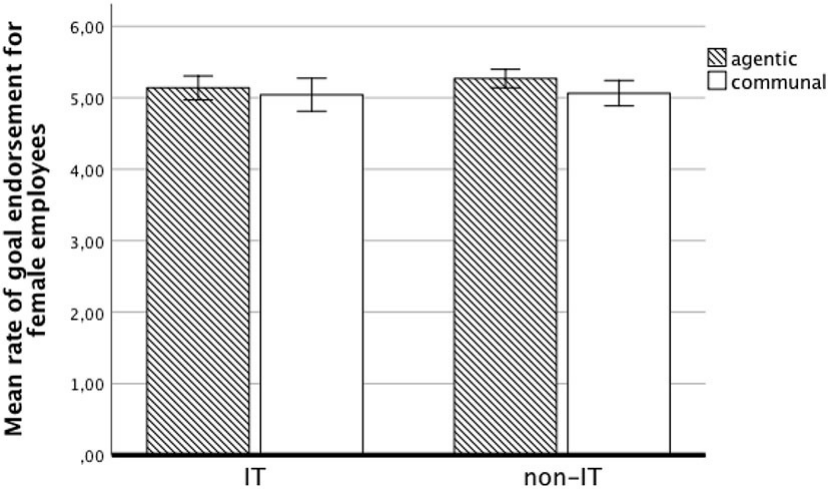
Mean rate of goal endorsement for female employees.

In the last step we compared female IT students and female IT employees in terms of goal endorsement. The difference was not significant for agentic goals [F _(1,153)_ = 0.03, *p* = 0.858, η^2^ = 0.01], but was significant for communal goals [*F*_(1,153)_ = 16.13, *p* < 0.001, η^2^ = 0.10]: female IT employees had higher communal goal endorsement than did the students. Differences in goal endorsement between women in IT and non-IT field (both students and employees) are presented in Figures 1, 2.

The same pattern was for observed for men: male IT employees rated agentic goals at the same level as male students [*F*_(1,175)_ = 0.11, *p* = 0.741, η^2^ = 0.01] however, they had higher communal goal endorsement than did the students [*F*_(1,175)_ = 12.59, *p* < 0.001, η^2^ = 0.07].

### Goal affordance

First, we compare women in IT to men in IT in terms of goal affordance (Hypothesis 2a and 2b). MANOVA with communal goal affordance as a dependent variable showed no significant differences between female and male employees [*F*_(1,203)_ = 2.92, *p* = 0.089, η^2^ = 0.01] and students [*F*_(1,127)_ = 0.22, *p* = 0.693, η^2^ = 0.01]. The difference for agentic goal affordance as a dependent variable was not significant between male and female IT employees [*F*_(1,203)_ = 1.48, *p* = 0.225, η^2^ = 0.01] but was significant for male and female IT students [*F*_(1,127)_ = 9.15, *p* = 0.003, η^2^ = 0.07]: female students in comparison to male students more strongly perceived IT as allowing them to pursue agentic goals.

In the next step we compared IT and non-IT female professionals in terms of goal affordance (Hypothesis 2c and 2d). Analysis shows no significant difference between female employees in IT and in non-IT regarding communal goal affordance [*F*_(1,217)_ = 2.77, *p* = 0.097, η^2^ = 0.01]. A different pattern was observed for students: female students in IT have lower communal goal affordance than non-IT students [*F*_(1,128)_ = 5.24, *p* = 0.024, η^2^ = 0.04].

We also compared IT and non-IT male professionals. For communal goal affordance, the difference for employees was not significant [*F*_(1,206)_ = 0.99, *p* = 0.320, η^2^ = 0.01], but it was for students [*F*_(1,102)_ = 9.54, *p* = 0.003, η^2^ = 0.09]. As in the case of female, male students in IT have lower communal goal affordance than non-IT students.

For agentic goal affordance, the difference was significant for female employees [*F*_(1,217)_ = 5.46, *p* = 0.020, η^2^ = 0.03]and students [*F*_(1,128_) = 8.26, *p* = 0.005, η^2^ = 0.06]: both IT female employees and students have higher agentic goals affordance than non-IT employees and students in the comparison group. For male employees, the difference in agentic goal affordance was not significant [*F*_(1,206)_ = 0.08, *p* = 0.774, η^2^ = 0.01]. The same lack of effect was for male students [*F*_(1,102)_ = 0.45, *p* = 0.505, η^2^ = 0.01].

Lastly, we compared female IT students and employees. Female students in IT have higher agentic goal affordance than employees [*F*_(1,153)_ = 48.32, *p* <0.001, η^2^ = 0.24]. The opposite pattern was for communal affordance: female IT employees have a stronger belief that the domain allows them to pursue communal goals [*F*_(1,153)_ = 10.30, *p* = 0.002, η^2^ = 0.06] than do the female students. Male students in IT have higher agentic goal affordance than do the male employees [*F*_(1,175)_ = 17.49, *p* <0.001, η^2^ = 0.09], but this effect was not observed for communal congruency [*F*_(1,175)_ = 2.79, *p* = 0.097, η^2^ = 0.02].

### Sense of social belonging

First, we compared female and male employees in IT (Hypothesis 3a). The effect was significant [*F*_(1,203)_ = 7.16, *p* = 0.008, η^2^ = 0.03]: contrary to our hypothesis, women in IT had higher sense of belonging to the field than men, but this effect was not significant for students [*F*_(1,125)_ = 0.87, *p* = 0.352, η^2^ = 0.01].

The comparison of females in IT to those in the control group shows no difference for employees [*F*_(1,217)_ = 2.95, *p* = 0.087, η^2^ = 0.01] and students [*F*_(1,128)_ = 1.96, *p* = 0.164, η^2^ = 0.01]. There was also no significant effect for male employees [*F*_(1,206)_ = 0.08, *p* = 0.773, η^2^ = 0.01] and male students [*F*_(1,102)_ = 3.65, *p* = 0.059, η^2^ = 0.04].

Female employees in IT have a higher sense of belonging to the field than do the students [*F*_(1,153)_ = 16.16, *p* <0.000, η^2^ = 0.10). There was no such effect for men in IT [*F*_(1,175)_ = 0.39, *p* = 0.531, η^2^ = 0.01].

### Self-efficacy

Contrary to our hypothesis 4a, female employees in IT had the same level of self-efficacy as male employees [*F*_(1,203)_ = 0.33, *p* = 0.566, η^2^ = 0.01]. What is interesting, this effect was not true for students [*F*_(1,125)_ = 4.77, *p* <0.031, η^2^ = 0.04]. Confirming our hypothesis, female students in IT had lower levels of self-efficacy than male students.

Moreover, women in IT had the same level of self-efficacy as women in the non-IT field, as was true for employees [*F* (1,217) = 0.01, *p* = 0.993, η^2^ < 0.01] and students [*F* (1,128) = 1.80, *p* = 0.182, η^2^ = 0.01]. There was no effect for male employees [*F*_(1,206)_ = 0.56, *p* = 0.455, η^2^ < 0.01] and male students [*F* (1,102) = 0.01, *p* = 0.966, η^2^ < 0.01].

What is interesting, female employees in IT had significantly higher self-efficacy than IT female students [*F*_(1,153)_ = 56.22, *p* <0.001, η^2^ = 0.27]. This effect was also observed for men in IT [*F*_(1,175)_ = 16.41, *p* <0.001, η^2^ = 0.09].

Data presented in this article are available here https://osf.io/pfq8s/?view_only=10c957f6632d42f49e83c38bee0d94e7.

## General discussion

The European Commission and other world organizations are raising the alarm about the shortage of staff in the IT sector (European Commission, 2008; United Nations Technology and Innovation Labs Report, 2019; Eurofound, 2021). The inequality in terms of staff's gender is also a serious concern in high tech innovative companies since considerable amount of data show that adequate female representation in the workplace contributes to better outcomes of the company (Hunt et al., 2015).

In our study we sampled men and women in the IT sector on different levels of their professional life (students and employees). Our goal was to study the possible gender differences in various aspects of the perception of the IT domain to inform potential interventions aiming at retaining women, who are already educated in this domain, in the IT sector. We expected to find specific gender differences and differences between IT and non-IT individuals. We focused on their goal endorsement and affordance, sense of social belonging, and self-efficacy. We were also interested in exploring the differences between students and women and men already employed in a given domain.

### Women and men within IT and outside of IT

To compare women and men within IT and outside of IT, we examined the goal endorsement and affordance and tested for potential gender and group differences. We predicted that women in IT will value communal goals more than men in IT (H 1a), but less so than women in the non-IT sector (H 1b). Only the first assumption turned out to be true. Women in our IT employee sample indeed valued communal goals more than men yet they valued them equally as women in the non-IT group. Although it is important to note that this gender difference was not so prominent, with the men valuing communal goals only marginally less than women. This result is consistent with other findings showing that for women communal goals play an important role (Pyrkosz-Pacyna et al., 2019) and may in turn predict their engagement in specific lines of work, for example in the STEM fields (Cheryan et al., 2009). Our results also show that the opportunity to realize one's communal goals might be important for men in IT as well. However, we also did not show that women in IT care less about communal goals than women in other professions (H 1c). Therefore, perceiving IT as a domain where achieving communal goals is possible might be one of the factors contributing to greater female representation in IT but it can also appeal to men, as they, while pursuing work-life balance, might be also finding communal goals as appealing (Diekman et al., 2010; Croft et al., 2015; Steinberg and Diekman, 2018). We also wanted to see whether communal goals are more important than agentic goals for women in IT (H 1d). On the one hand, women in general tend to value communal goals slightly more than agentic goals (Wood and Eagly, 2002). On the other hand, perhaps women who choose IT do so because of their particular focus on agentic goals. We predicted that the first explanation is the more plausible one, yet our findings showed that women in IT valued agentic goals no less than they value communal goals. People stereotypically tend to think that women care less about agentic goals and simply strive for career development less than they care about reaching communal goals. Our results align with the increasing bulk of literature showing that agentic goals are becoming equally important for both women and men (Moore et al., 2008; Pyrkosz-Pacyna et al., 2019). We may thus suspect that women might be actually attracted to IT by perceiving it as allowing their agentic goals to be fulfilled - our study participants in both IT and in the non-IT group, did value agentic goals as well as the communal ones. Recruitment or promotion strategies omitting this aspect of women's values can influence future decisions regarding their engagement in STEM.

In the next step we wanted to see whether women in IT perceive their field as enabling them to achieve their goals. We hypothesized that women in IT will perceive less communal goal affordance than do the men in IT (H 2a) and the women in the control group (H 2c). Neither of our hypotheses were confirmed, showing that there might be no gender or interdomain difference between women in the IT and non-IT sectors in terms of communal goal affordance. It is worth mentioning here that in general the level of perceived communal and agentic affordance in the studied sample was at the medium level. When it comes to agentic goal affordance, again our hypotheses were not confirmed - men and women did not differ in perception of agentic goal affordance in IT (H 2b). This is a positive finding in the light of the fact that lack of agentic goal affordance was in previous studies found to be contributing to women dropping out from STEM domains (Diekman et al., 2010). When aiming for interdomain comparison, women in IT described their domain as affording agentic goals to a higher extent than did the women in the non-IT group (H 2d). This result is in accordance with our hypotheses. Even though it might seem women in male dominated fields might be less able to achieve their agentic goals, in our sample this was not the case. This finding is also of great practical significance - since agentic goals are increasingly important to women, the perception of IT as affording this striving might increase the likelihood of choosing an IT career path.

As for the sense of social belonging we predicted that women in IT will have a lower sense of social belonging than do the men in IT (H 3a) and the women in the control group (H 3b). Again, our assumptions were not entirely supported. Surprisingly, and contrary to previous findings, we found that women have a higher sense of belonging to the field than do the men in IT. This result may indicate that the IT domain might have become more welcoming toward women especially in the light of the many interventions directed at women to encourage them to study IT. Several studies (Cheryan and Plaut, 2010; Good et al., 2012; Tellhed et al., 2017; Aelenei et al., 2020) did show that indeed women tend to feel that they do not naturally belong in STEM but if their sense of fit was experimentally increased then they declared higher interest in career in STEM. Such campaigns are recognized at technical universities across Poland (see: IT for SHE or Girls as Engineers! & Girls go Science! Campaigns) and they may have increased the visibility of women pursuing IT careers.

Efforts toward validating the effectiveness of interventions might shed a light on the changes that need to be implemented to increase their efficiency.

Finally, we focused on women's vs men's assumed lower self-efficacy. Yet again in our study we did not find any significant gender or interdomain differences, thus proving that women in IT have a self-efficacy similar to that of men in IT (H 4a) and as that of women in the comparison group (H 4b).

Taken together, as shown by these results, we were unable to show many of the anticipated gender differences that might prove that there are in fact fewer gender-based differences than is assumed. Men and women in IT seem to be alike in terms of their perception of goal affordance, sense of belonging, and self-efficacy. In fact, in the IT sample, the women even outscored men in terms of the sense of social belonging to their workplace. All these findings, in our opinion, support a strong need for continuous monitoring of the gender (in)balance to be able to design and implement the interventions that address specific problems with appropriate, evidence-based solutions.

### Employees and students within IT

Our second line of analysis focused on exploring similarities and differences among female IT employees and students. Our data showed that in the study sample female IT students valued communal goals to a lesser degree than do women already working in IT. The same effect was also visible for male IT workers and students, with male employees valuing communal goals significantly higher than do the IT students. When it comes to agentic goals, we found that they were equally important for all groups, namely IT male and female workers and students, and in all cases more so than communal goals. These results indicate that there are noticeable differences in goal endorsement on different career levels in terms of communal goals - they seem to be more important for employees than they do for students. This difference might be due either to generational factors or to a change in attitude over time, which would be possible to examine only with longitudinal research. We argue that this result is reflecting the current focus of students, both male and female, which is to achieve agentic work goals. Only when these goals are secured employees start to focus on communal goals, perhaps due in part to work conditions that require tapping into these competences, i.e. cooperation, teamwork or communication. Our results are congruent with some results present in the literature concerning IT employees indicating that agentic goals are becoming equally important for men as well as women (Moore et al., 2008; Pyrkosz-Pacyna et al., 2019) and thus recruitment or promotion strategies failing to underline the agentic aspect of women's values, along with communal ones, might not be fully effective in attracting female candidates.

We also found a difference between students and employees in terms of their perception of goal affordance. Women already working in IT found that it better afforded the attainment of communal goals than did the IT students. The opposite was true for agentic goals affordance. These results show that again, there is a significant difference in perception of the IT domain by those already familiar with it and those yet to be employed there.

Taken together, female IT students value agentic goals more than they value communal goals (contrary to common gender stereotypes) and at the same time perceive greater possibility to achieve those goals in their future workplace than do the men in IT and the women in the comparison group. Although research within STEM shows that STEM occupations are perceived as affording communal goals to a lesser extent that other professions (see e.g., Diekman et al., 2010) the IT sector may signalize that communal goal congruence is achievable among female employees (Herz, 1997; Sanger et al., 1997; Clayton et al., 2009; Santos et al., 2022).

These results should indicate a higher probability of women to engage in IT after graduation. They also suggest that information on goal attainment in a given workplace might be a relevant practical strategy to communicate with potential candidates. At the same time, monitoring the goal strivings and goal attainment among men and women already working in IT might provide a significant advantage when it comes to achieving gender balance in this sector.

When it comes to social belonging, female IT students revealed levels similar to those of their male colleagues and the female students in the comparison group. It would appear that once women get into IT, they tend to enjoy a similar level of sense of social belonging than do the other study participants. We did however find a significant gender difference when it comes to IT student's self-efficacy, namely that women have considerably lower self-efficacy than men. Lower self-efficacy than that among men might be a crucial ingredient when searching for the origins of female underrepresentation in IT. Many studies thus far have shown how low self-efficacy may influence decisions regarding future career goals in the context of women in STEM (Correll, 2001; Singh et al., 2007; Good et al., 2012; Sterling et al., 2020; Stewart et al., 2020). It is important to note that we have found no difference in self-efficacy among female students in IT and those in the comparison group. We suspect that IT students might reveal lower self-efficacy, as they are subjected to constant comparison with male counterparts and various forms of gender stereotypes. However, this turned out to be a false assumption. Also, it is important to note, that the self-efficacy gender difference was not present among the employees sample. This indicates that the highly vulnerable period for women when it comes to building self-efficacy is during the process of gaining education. This result highlights that more attention to building self-efficacy among IT students and longitudinal research would be recommended to allow for more precise insights into factors contributing to lower female representation in STEM over time.

Based on our findings and previous literature we prepared a list of practical implications to be taken into consideration for policymakers and individuals involved in the process of designing and delivering gender diversity in STEM interventions.

### Practical implications for policymakers and interventions designers

#### Tailoring problem-focused interventions for different stages of education and career

Our study showed that there is a significant difference between male and female IT students in terms of self-efficacy, namely the self-efficacy among female IT students is lower than among male students. This effect was not however found among IT employees suggesting that different interventions are due for different moments in one's career trajectory. For students more attention to building their self-efficacy would be needed whereas less so for employees.

#### Importance of monitoring of longitudinal effects

Our research showed that goal endorsement among students and employees is different. For example, we discovered that communal and agentic goal endorsement is equally important for IT female employees but not for IT female students—for IT female students agentic goals are significantly more important. Again, these results call for different intervention strategies but also, they point out to the importance of conducting longitudinal research focused on women in STEM fields. Longitudinal research enables more precise insights into factors contributing to lower female representation in STEM over time.

#### Taking into account socio-economic and geographical context of intervention beneficiaries

Most studies regarding women in STEM are conducted in western cultures. There is also considerable literature regarding women in STEM professions in developing economies (e.g., Stoet and Geary, 2018; Huang et al., 2020). These studies point out to some important differences among studied mechanisms for example Stoet and Geary (2018) using international database of over half a million adolescents' achievement in science, mathematics and reading showed that sex differences in the academic readiness to pursuit STEM careers favoring boys increase with gender equality levels. It is therefore important to foster more research including various country-level variables and economic contexts to find and test solutions suitable for specific contexts.

#### Providing women with insights into the specifics of pursuing an IT career

As our research showed, the perception of work in IT is in some respects significantly different among female students and female employees. Therefore, interventions aimed at showcasing careers in various STEM disciplines, including IT, would be beneficial for acquiring an accurate and informed view on the matter. Such interventions may include among others: guest lectures, open days, mentoring programs, video testimonials, internships, and other. Especially mentoring has been proven to be an effective tool in supporting women in STEM (Dennehy and Dasgupta, 2017).

#### Investigating and pilot testing various interventions aimed at boosting self-efficacy

Our research, as well as numerous others, showed that women tend to have lower levels of self-efficacy than men. The influence of low self-efficacy on educational and vocational decisions is well documented (e.g., Dennehy and Dasgupta, 2017; Easterbrook et al., 2021). Specifically in highly male-dominated fields women's low self-efficacy is of vast importance. Therefore, it is crucial to design and implement interventions aimed at raising the level of self-efficacy among women, especially students since as our study showed, the gender difference seems to not appear among working individuals as they do among students.

#### Continuous monitoring of gender related mechanisms in STEM fields

Much valuable research to this date highlighted the importance of social belonging when it comes to female representation in STEM (e.g., Cheryan and Plaut, 2010; Diekman et al., 2010). These results emphasize the value of both theory and evidence-based intervention aimed at attracting and keeping women in STEM. Since these interventions are strongly contextualized, for example by time and target group, even when proving effective in one context might not be as effective in others (Easterbrook et al., 2021). Hence, they need to be tailored by focusing on the needs of the given target group in a given time and based on proper diagnosis of the needs of the group at which the intervention is aimed. With development of gender equality plans in different education contexts currently happening across academic institutions across the world new measures and new goals might have to be established to improve gender balance in different fields. For example, in our study we hypothesized that women in the IT occupation will have a lower sense of social belonging than men in similar positions. This assumption turned out not to be true. Quite the opposite - surprisingly, in our sample women in IT positions had a higher sense of social belonging than men. Results that we gathered suggest that at least in some cases this effect might be currently less pervasive. In our opinion continuous replication of such culturally sensitive mechanisms would be beneficial for achieving more accurate and timely data.

### Limitations and future research

In general, our findings highlight two important factors relating to women's presence in the IT sector. First, one cannot generalize the results obtained among students to the employee population. We found considerable differences in almost all the measured aspects when comparing students and employees' samples within IT. Second, the results indicate that there might be a potential shift in perception of IT employment when moving up the occupational ladder. Future research in this area might be very useful in the effort to establish whether there is an actual change of goals and beliefs. A longitudinal study paradigm would be especially beneficial for further analysis especially in terms of investigating if the discovered differences between researched groups stem from changes in experience or are generational differences or even a result of comparing two distinct age groups.

Our study is not free from limitations. Firstly, the participants of our study were recruited from two IT companies, so our findings might simply reflect the given companies' focus on the inclusion of women. Indeed, both companies avow such policies. Furthermore, the choice of banking and management as comparative fields does not allow for a full comparison with the non-STEM field. Secondly, even though the underrepresentation of women in IT is rather universal country-wise, we do observe some country-level variations. A recent study focusing on 41 countries in the OECD and EU shows that the percentage of women working in the information and communication technology sector varies from 9 to 11% in countries with lowest representation of women in IT (Slovak Republik, Turkey and Israel) up to maximum 24-30% in countries with the highest representation of women in IT (Bulgaria, Australia, and Romania; Honeypot, 2018). In a study by Stoet and Geary (2018), the authors point out the paradox of less developed countries having a surprisingly high number of women in STEM, presumably due to the perception of STEM as providing good socio-economic improvement possibilities. As in many other cases, the vast preponderance of research related to lower representation of women in STEM is conducted in the US and other developed countries. As this has been conducted in Poland, a country with relatively little experience with inclusion programs within IT companies and very traditional when it comes to gender stereotypes, our study contributes to the understanding of IT representation within a broader cultural sample. In terms of methodological improvements in future research measures, specifically addressing work in IT (in our study we asked for participants' line of work) might be more accurate.

## Conclusions

Our findings contribute to current research lines focused on lower representation in the IT sector by adding another under-researched context of Poland (most of the current studies are conducted in the US or in other western countries). Although there is an abundance of job offers and career possibilities in IT for both men and women, companies struggle to attract women to IT positions. The practical implication of our study is the broadened understanding of the factors contributing to the low representation of women in IT, specifically in terms of the goal congruence perception with its impact on the sense of belonging and thus on the sense of engagement with work. Our results show that women might feel welcomed and included in the IT sector.

Gender diversity translates to a better fit between technology and society. That women have almost no voice in the creation of some important technological innovations is detrimental to the industry and society (Selby et al., 1997). Yet, 88 % of information technology patents are invented by male-only teams. Lastly, the IT industry is one of the best paying in the economy (Hays, 2020). Consequently, low female representation in IT contributes to an elevated gender pay gap Fry et al. (2021). Studies show that the gender pay gap in STEM does exist although it is smaller than in other sectors: 14 % in STEM vs. 20 % in non-STEM fields (Beede et al., 2011; Jasko et al., 2020), which contributes to a narrowing of the pay gap ratio. Some researchers argue that the pay gap might be even larger in IT due to persisting stereotypes and various well-known mechanisms such as the sticky floor effect - discriminatory employment pattern keeping mainly women in the lower positions with low mobility and invisible barriers to their career advancement (Segovia-Pérez et al., 2020). And lastly, IT is perceived rather favorably by the IT workforce as a good place to work, also by women - in the study by Hewlett et al. (2008) 80 % of women report ‘loving their work'. Our results also seem to confirm the assertion which should be disseminated more widely to attract more women within IT. They do like it. They do feel they belong there.

## Data availability statement

The datasets presented in this study can be found in online repositories. The names of the repository/repositories and accession number(s) can be found in the article/supplementary material.

## Ethics statement

Ethical review and approval was not required for the study on human participants in accordance with the local legislation and institutional requirements. Written informed consent for participation was not required for this study in accordance with the national legislation and the institutional requirements.

## Author contributions

KD organized the database and performed the statistical analysis. JP-P wrote the first draft of the manuscript. NK-B provided essential corrections to the sections of the manuscript. All authors contributed to the conception and design of the study, manuscript revision, read, and approved the submitted version.

## Conflict of interest

The authors declare that the research was conducted in the absence of any commercial or financial relationships that could be construed as a potential conflict of interest.

## Publisher's note

All claims expressed in this article are solely those of the authors and do not necessarily represent those of their affiliated organizations, or those of the publisher, the editors and the reviewers. Any product that may be evaluated in this article, or claim that may be made by its manufacturer, is not guaranteed or endorsed by the publisher.
